# Resection of a giant paravertebral retroperitoneal schwannoma via the weaver approach: a case report on technical refinements and surgical considerations

**DOI:** 10.3389/fonc.2026.1778891

**Published:** 2026-04-29

**Authors:** Jun Shen, Qian An, Sufen Wang, Ziji Li, Zihao Zhou, Shaolin Zhang

**Affiliations:** 1 Department of Neurosurgery, The First Affiliated Hospital of Wannan Medical College (YiJiShan Hospital of Wannan Medical College), Wuhu, Anhui, China; 2 Department of Respiratory and Critical Care Medicine, Wuhu Hospital of Traditional Chinese Medicine, Wuhu, Anhui, China; 3 Department of Pathology, The First Affiliated Hospital of Wannan Medical College (YiJiShan Hospital of Wannan Medical College), Wuhu, Anhui, China

**Keywords:** intervertebral, paravertebral, retroperitoneal, schwannoma, weaver approach

## Abstract

**Objective:**

This study aims to evaluate the surgical feasibility, technical refinements, and clinical outcomes of resecting a giant paravertebral retroperitoneal schwannoma using the Weaver intermuscular approach. It also emphasizes key anatomical considerations, surgical techniques, and potential pitfalls to guide spine and peripheral nerve surgeons in managing similarly complex lesions.

**Case description:**

A 51-year-old woman presented with a six-month history of progressive discomfort in the left lumbar region. Physical examination revealed no focal neurological deficits or other significant findings. Magnetic resonance imaging revealed a large left-sided paravertebral retroperitoneal mass measuring 90 × 108 × 122 mm, extending through the L3–L4 intervertebral foramen and compressing adjacent structures, including the abdominal aorta, kidney, and psoas muscle. Radiological findings suggested a neurogenic tumor. The tumor was completely resected using a posterior muscle-splitting Weaver approach. Key technical refinements included precise preoperative imaging-based trajectory planning, partial resection of the L3 and L4 transverse processes to expand the lateral working corridor, staged intracapsular debulking, meticulous extracapsular dissection, and preservation of retroperitoneal integrity.

**Conclusions:**

This case demonstrates that the Weaver intermuscular approach can be safely and effectively applied to giant paravertebral retroperitoneal schwannomas with foraminal extension. With appropriate patient selection and meticulous microsurgical technique, this posterior muscle-sparing approach offers a viable alternative to traditional anterior or combined approaches, minimizing surgical morbidity and preserving spinal stability.

## 1 Introduction

Schwannomas involving the retroperitoneal space are rare, accounting for approximately 1% to 3% of all schwannomas. Their deep location and nonspecific clinical presentation pose considerable diagnostic and surgical challenges (1). Retroperitoneal schwannomas often present as large, slow-growing masses that remain asymptomatic until they reach a considerable size, at which point compression of adjacent structures may cause vague symptoms such as abdominal discomfort, pain, or gastrointestinal disturbances (2, 3). Because of their size and proximity to major vascular and visceral structures, complete surgical excision remains the treatment of choice to prevent local recurrence (1). Tumors that extend into the paravertebral retroperitoneal space or closely abutting vital neurovascular structures are even rarer and add an additional layer of complexity. Traditional open surgical approaches for retroperitoneal schwannomas—such as anterior transabdominal, posterior midline, or combined techniques—have been employed; however, each method presents inherent challenges, including extensive soft tissue dissection, increased blood loss, and prolonged recovery (4). Minimally invasive approaches (laparoscopic or retroperitoneoscopic) have been successfully described for select retroperitoneal tumors, offering reduced morbidity; however, these techniques may still be limited by tumor size, location, and the surgeon’s familiarity with deep retroperitoneal anatomy (5, 6).

The Weaver approach, originally described for exposure of the posterior elements and the lateral paraspinal region, allows access through the paraspinal muscle interval without extensive disruption of bone or soft tissue. This approach has been successfully employed for lumbar disc herniations and the placement of pedicle screw instrumentation (7–9). However, there is a paucity of literature describing the application of the Weaver approach for large paravertebral retroperitoneal schwannomas of the scale presented here. This gap is significant, as such tumors often pose technical challenges that may be uniquely addressed by the anatomical corridor provided by the Weaver approach. In the present case, we report the successful resection of a giant paravertebral retroperitoneal schwannoma measuring 90 × 108 × 122 mm using the Weaver approach. This case highlights critical technical refinements, multidisciplinary surgical considerations, and postoperative outcomes. By examining this rare entity within the context of existing surgical strategies for retroperitoneal schwannomas, we aim to provide practical insights for spine and peripheral nerve surgeons managing similarly complex lesions.

## 2 Case presentation

A 51-year-old woman was admitted with a six-month history of progressive, dull pain and distension in the left lumbar region. The symptoms initially developed without any apparent trigger and were intermittent. She denied experiencing numbness or weakness in the lower extremities, and the discomfort was partially relieved by rest. Consequently, she did not seek medical attention at that time. One month prior to admission, the lumbar discomfort gradually worsened and became persistent, no longer alleviated by rest, prompting her to seek evaluation at our outpatient clinic. The patient had a body mass index (BMI) of 38.1 kg/m². Physical examination revealed no focal neurological deficits or other significant findings.

**Figure 1:**
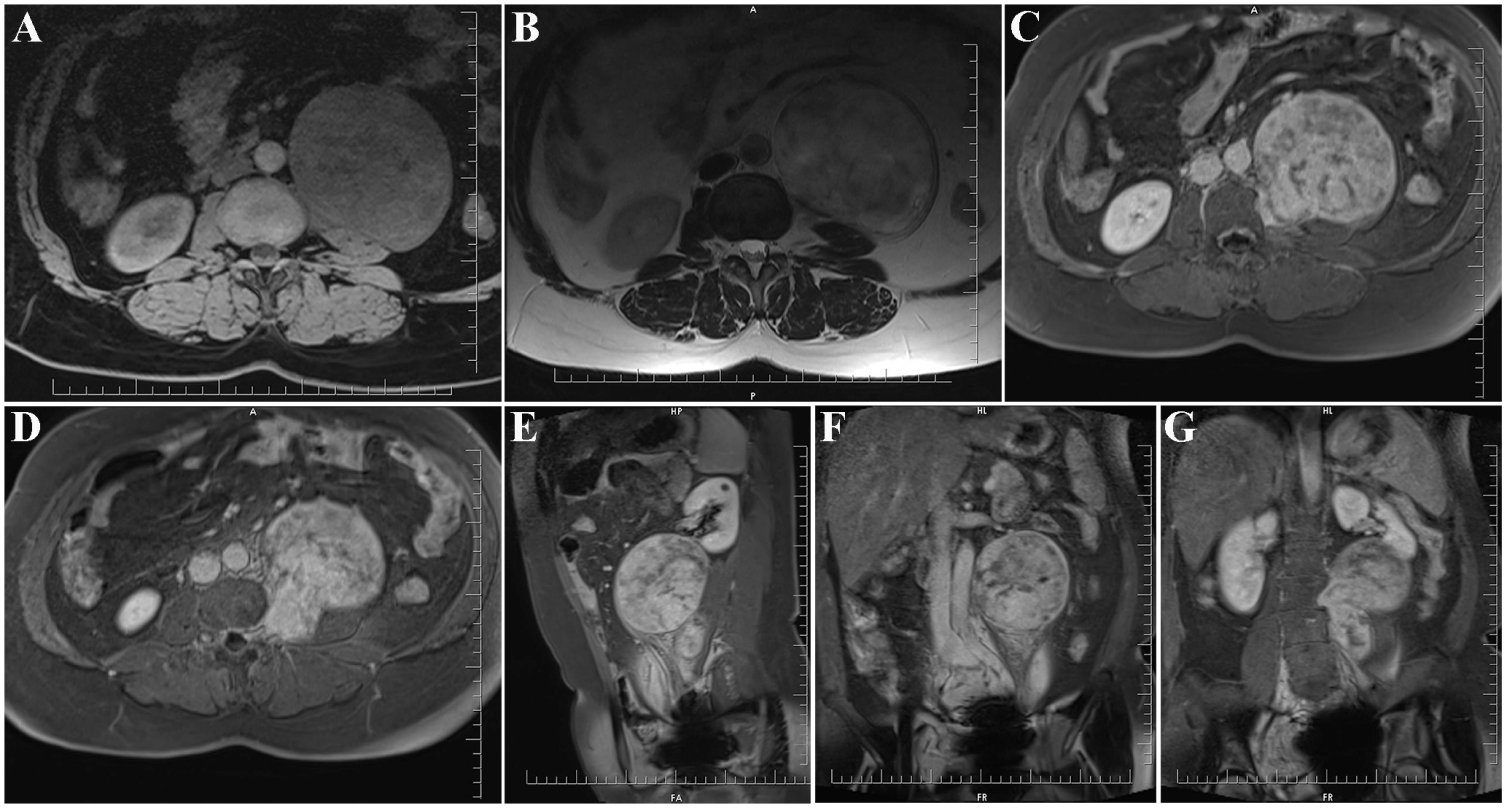
Preoperative MRI of the patient. **(A)** Axial T1-weighted image showing the tumor with isointense signal intensity. **(B)** Axial T2-weighted image demonstrating heterogeneous hyperintensity of the tumor. **(C)** Axial contrast-enhanced T1-weighted image revealing marked heterogeneous enhancement. **(D)** The tumor originates from the left L3–4 intervertebral foramen. **(E)** Sagittal contrast-enhanced T1-weighted image. **(F)** Coronal contrast-enhanced T1-weighted image showing the tumor closely abutting the abdominal aorta. **(G)** Coronal contrast-enhanced T1-weighted image demonstrating close contact between the tumor and the kidney and ureter, with superior displacement of the kidney.

Lumbar magnetic resonance imaging (MRI) revealed an irregularly shaped mass in the left retroperitoneal space with well-defined margins. The lesion measured approximately 90 × 108 × 122 mm (left–right × anteroposterior × craniocaudal). It appeared isointense on T1-weighted images (**Figure 1A**) and heterogeneously hyperintense on T2-weighted images (**Figure 1B**), with marked heterogeneous enhancement following contrast administration (**Figures 1C–E**). Notably, a focal “beak-like” extension of the lesion into the left L3/4 intervertebral foramen was observed (**Figure 1D**). The L3 vertebral body showed compression and concavity. The tumor was closely adjacent to the abdominal aorta (**Figure 1F**) and caused displacement of the left psoas major muscle and left kidney (**Figure 1G**). Based on imaging findings, a neurogenic tumor was suspected.

**Figure 2:**
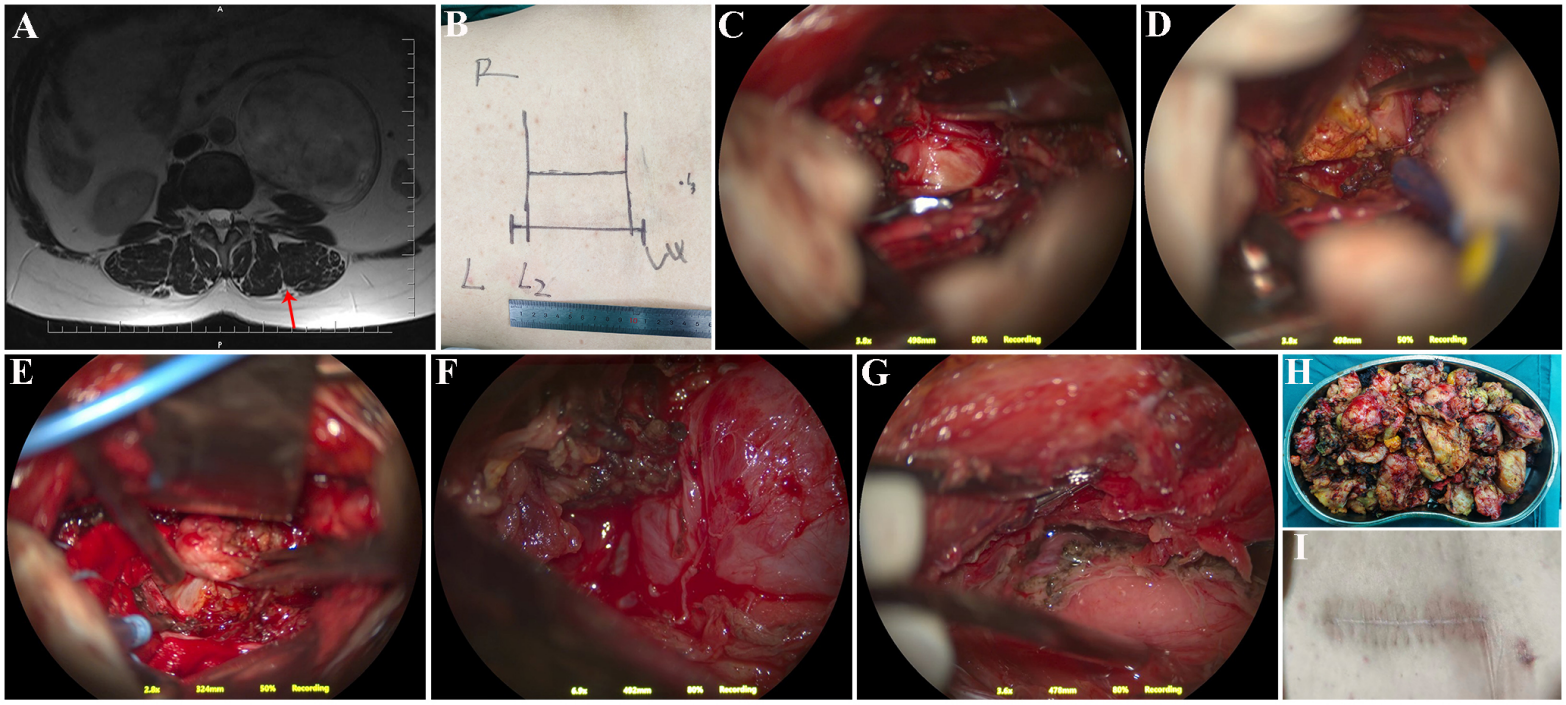
Surgical procedure. **(A)** Planned surgical trajectory; the red arrow indicates the Weaver intermuscular plane. **(B)** Skin incision. **(C)** Tumor exposure after opening the intertransverse ligament. **(D)** Intratumoral piecemeal debulking. **(E)** Careful dissection between the tumor and the retroperitoneum. **(F)** Final removal of the intervertebral foramen and paravertebral components of the tumor. **(G)** Complete tumor resection with preservation of the intact retroperitoneum. **(H)** Piecemeal-resected tumor specimens. **(I)** Surgical incision after suture removal.

The surgical plan involved exposing and removing the tumor using the Weaver approach (**Figure 2A**). After induction of stable general anesthesia, a urologist assisted with the placement of a left ureteral catheter and a urinary catheter. Under C-arm fluoroscopic guidance, the L2-L4 vertebral levels were identified. A longitudinal skin incision approximately 11 cm in length was made 4 cm lateral to the midline, extending along the line connecting the L2-L4 vertebrae (**Figures 2B, I**). The skin and lumbar fascia were incised, and blunt dissection was performed through the intermuscular plane between the longissimus and iliocostalis muscles to expose the transverse processes of L2, L3, and L4. A retractor was applied to maintain exposure. The muscular attachments over the transverse processes of L2-L4 were detached, and the intertransverse ligament at the L3-L4 level was opened. Partial resection of the L3 and L4 transverse processes was performed using a high-speed drill. Intraoperatively, the tumor was found to originate from the left L3-L4 intervertebral foramen and extend anteriorly into the retroperitoneal space (**Figure 2C**). The mass appeared yellowish-white in color, firm in consistency, and moderately vascular. Initial intracapsular piecemeal debulking was performed to reduce tumor volume (**Figure 2D**). As the tumor gradually decreased in size, careful dissection was carried out along the tumor capsule to separate it from the surrounding normal tissues (**Figure 2E**). The tumor was densely adherent to the posterior peritoneum, which was meticulously dissected while preserving the integrity of the retroperitoneal structures. Ultimately, the tumor within the L3-L4 intervertebral foramen, as well as the paravertebral components at the L2-L3 and L4-L5 levels (**Figure 2F**), were completely excised under microscopic visualization, achieving gross total resection (**Figures 2G, H**).

**Figure 3:**
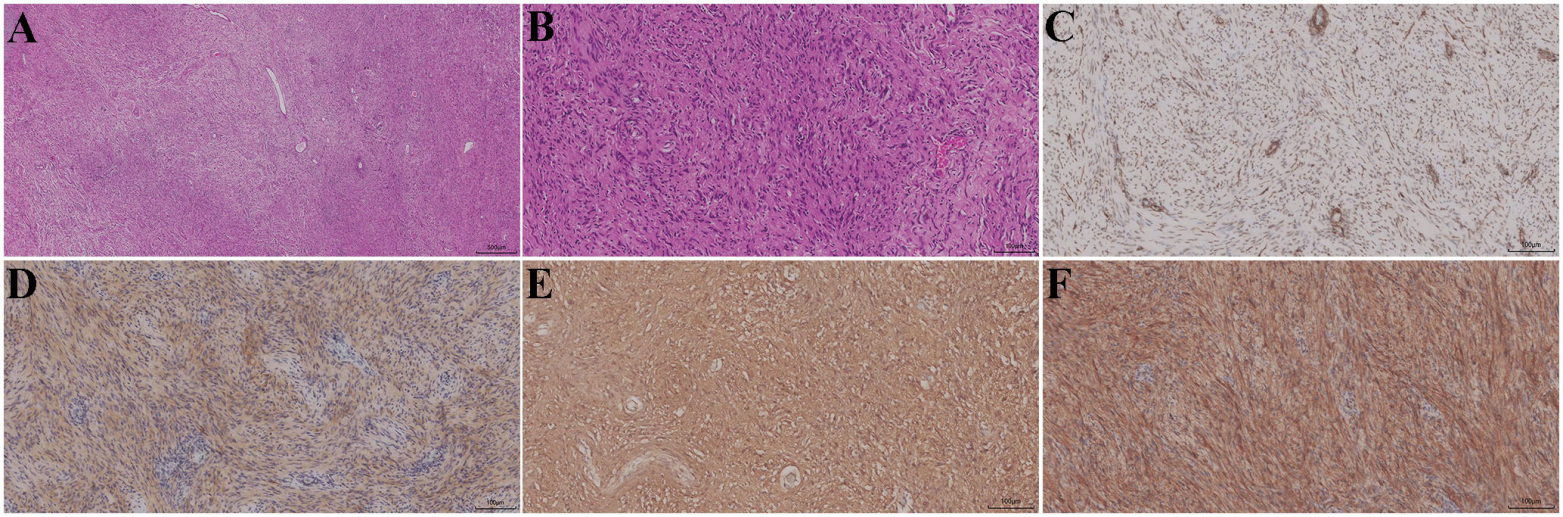
Histopathological findings. **(A)** Low-power microscopic view. **(B)** High-power microscopic view. **(C)** Nuclear positivity for smooth muscle actin. **(D)** Positivity for epithelial membrane antigen. **(E)** Strong positivity for S-100 protein. **(F)** Cytoplasmic positivity for β-catenin.

Histopathological examination revealed a well-circumscribed lesion at low magnification, composed of spindle-shaped tumor cells with alternating Antoni A (hypercellular) and Antoni B (hypocellular) areas. The tumor cells were arranged in palisading, interlacing, and whorled patterns (**Figure 3A**). At high magnification, the tumor cells exhibited indistinct cell borders, elongated spindle-shaped nuclei with fine chromatin, and small nucleoli. The stroma contained scattered lymphocytes and foamy histiocytes, with focal lymphoid aggregates and areas of hyalinized vascular changes (**Figure 3B**). Immunohistochemical staining demonstrated that the tumor cells were negative for AE1/AE3, desmin, CD34, and progesterone receptor; partially positive for nuclear smooth muscle actin (**Figure 3C**) and epithelial membrane antigen (**Figure 3D**); strongly positive for S-100 protein (**Figure 3E**); positive for cytoplasmic β-catenin (**Figure 3F**); and showed a low proliferative index, with Ki-67 positivity of approximately 1% (**Figure 3G**). Based on the histological and immunohistochemical findings, the final pathological diagnosis was schwannoma.

**Figure 4:**
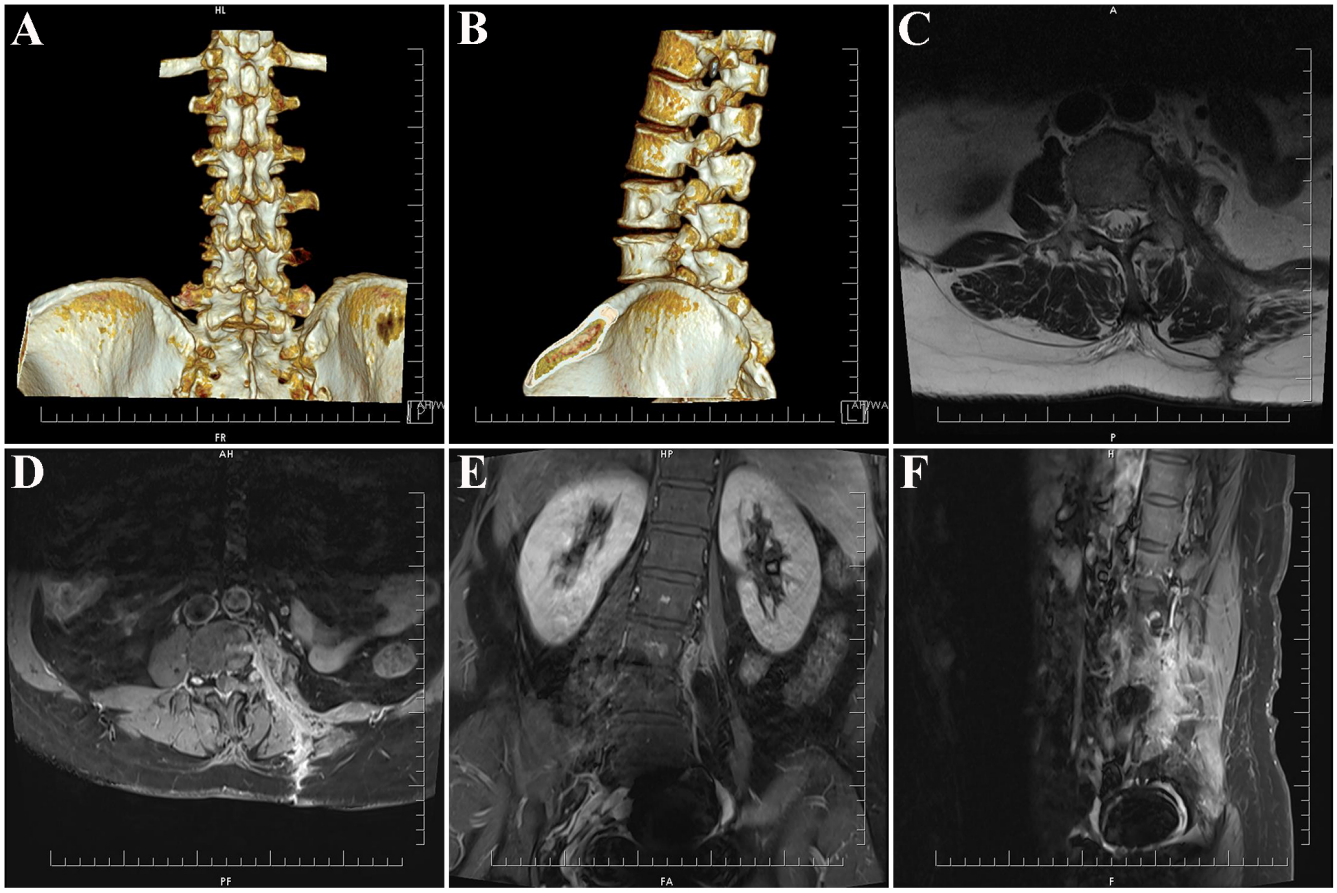
Postoperative three-dimensional CT and three-month follow-up MRI. **(A, B)** Three-dimensional CT reconstruction showing partial resection of the left L3 and L4 transverse processes, with preservation of other bony structures. **(C)** Postoperative T2-weighted image demonstrating the surgical corridor through the Weaver intermuscular plane. **(D–F)** Postoperative contrast-enhanced T1-weighted axial, coronal, and sagittal images confirming complete tumor resection.

Postoperatively, the patient exhibited no neurological deficits. Three-dimensional computed tomography (CT) confirmed partial resection of the left L3 and L4 transverse processes, with preservation of all other bony structures (**Figures 4A, B**). At the three-month follow-up, lumbar MRI demonstrated complete tumor resection with no evidence of recurrence (**Figures 4C–F**).

## 3 Discussion

Paravertebral retroperitoneal schwannomas are rare benign tumors that originate from the spinal nerve sheaths. They are often characterized by insidious growth, large tumor size at diagnosis, and complex anatomical relationships with adjacent neurovascular and visceral structures (2, 10, 11). When these tumors extend through the intervertebral foramen into the retroperitoneal space, surgical resection becomes particularly challenging due to limited exposure, proximity to major vessels, and the need to preserve spinal stability (12–14).

Traditional posterior midline approaches often require extensive dissection of the paraspinal muscles, bilateral laminectomy, facetectomy, and, in some cases, instrumented fusion, particularly for large foraminal or dumbbell-shaped tumors (15, 16). Although these methods provide effective tumor exposure, they are associated with significant muscle injury, postoperative pain, prolonged recovery, and potential long-term spinal instability (17). In contrast, muscle-splitting techniques, such as the Wiltse and Weaver approaches, offer direct access to the paravertebral and foraminal regions while preserving the midline osteoligamentous structures (7, 18, 19).

The present case demonstrates the feasibility of using the Weaver intermuscular approach for the complete resection of a giant paravertebral retroperitoneal schwannoma measuring 90 × 108 × 122 mm. Despite the tumor’s large size, foraminal extension, and close proximity to the abdominal aorta and retroperitoneal organs, gross total resection was achieved without spinal destabilization or neurological deficits.

Most previously reported giant retroperitoneal or paravertebral schwannomas have been managed using anterior transperitoneal, retroperitoneal, or combined anterior–posterior approaches, especially when tumors exceed 8–10 cm in diameter (12, 20–22). Although these approaches provide wide exposure, they carry inherent risks, including visceral injury, vascular complications, postoperative ileus, and prolonged hospitalization (3). In recent years, minimally invasive techniques, including robot-assisted laparoscopic surgery via a retroperitoneal approach, have been increasingly utilized in the management of retroperitoneal tumors. These methods offer several advantages, such as reduced surgical trauma, enhanced visualization, and faster postoperative recovery. However, for giant tumors with paravertebral extension, as in the present case, open surgical approaches may still provide better exposure and facilitate safer resection (23).

Posterior-only approaches using the Wiltse muscle-splitting corridor have been increasingly reported for extraforaminal and paravertebral nerve sheath tumors, yielding favorable outcomes in selected cases (24, 25). However, the Weaver approach—which utilizes a more lateral intermuscular plane between the longissimus and iliocostalis muscles—provides a wider lateral working corridor, which is particularly advantageous for tumors with extensive retroperitoneal extension (7, 26). Compared with previously published technical notes by our group and others (24–26), the current case further expands the indications for the Weaver approach to include giant tumors with multilevel foraminal involvement and retroperitoneal expansion. This demonstrates that tumor size alone should not be considered an absolute contraindication to a posterior muscle-splitting approach when meticulous microsurgical technique is applied. Several technical refinements were critical to the successful outcome in this case. First, precise preoperative imaging analysis using contrast-enhanced MRI was essential to delineate the tumor’s relationship with the abdominal aorta, kidney, psoas muscle, and intervertebral foramina. This enabled accurate trajectory planning and minimized intraoperative uncertainty. Second, partial resection of the L3 and L4 transverse processes, rather than facetectomy, provided sufficient exposure to the tumor base while preserving spinal stability. This strategy avoided the need for internal fixation, consistent with biomechanical studies demonstrating that unilateral transverse process removal does not significantly compromise spinal integrity (27). Third, intracapsular debulking prior to extracapsular dissection was instrumental in reducing tumor tension and facilitating safe separation from the retroperitoneum and adjacent vascular structures. This stepwise reduction technique has been emphasized in the management of giant schwannomas to minimize traction-related nerve injury and bleeding (28). Fourth, because the tumor was closely attached to the kidney and ureter, preoperative placement of a ureteral catheter was beneficial to facilitate identification of the retroperitoneum during surgery, thereby avoiding injury to the ureter and kidney. Finally, microsurgical dissection under high magnification allowed clear identification of the tumor–nerve interface, enabling complete resection while preserving neural elements. The low Ki-67 index and classical Antoni A/B histological pattern further supported the benign nature of the lesion, justifying aggressive yet nerve-sparing excision. Attention should be given to the fact that, although the final diagnosis in this case was a benign schwannoma, the role of preoperative biopsy in retroperitoneal tumors must be carefully considered. In particular, for lesions with suspicious imaging features, biopsy can help differentiate benign schwannomas from malignant peripheral nerve sheath tumors. This distinction is clinically important because intracapsular debulking of malignant tumors may increase the risk of tumor dissemination and recurrence. Therefore, thorough preoperative evaluation is essential when malignancy cannot be excluded.

Several surgical pearls can be gleaned from this case. First, the Weaver approach provides a direct, muscle-sparing corridor to paravertebral and foraminal tumors, especially when lateral or retroperitoneal extension predominates. This approach minimizes muscle ischemia, reduces postoperative pain, and accelerates recovery. Second, tumor size should not be the sole determinant of the surgical approach. Instead, the direction of tumor growth, foraminal anatomy, and relationship with critical structures should guide surgical planning. Even giant tumors may be safely resected posteriorly when growth is predominantly lateral. Third, controlled intracapsular decompression is a key maneuver to reduce operative risk. Attempting en bloc resection of massive schwannomas increases the likelihood of vascular or neural injury and should be avoided. However, several pitfalls warrant attention. Inadequate exposure due to insufficient transverse process resection may lead to blind dissection and an increased risk of complications. Additionally, failure to recognize tight adhesions between the tumor capsule and the retroperitoneum may result in peritoneal violation or vascular injury. Meticulous dissection and readiness to adjust the surgical corridor are therefore essential. Nevertheless, long-term follow-up is essential to assess the risk of recurrence and to validate the long-term safety of this surgical approach.

In conclusion, this case reinforces the concept that minimally invasive posterior muscle-splitting approaches can be safely applied to complex and giant paravertebral schwannomas when performed by experienced surgeons. By preserving spinal stability and avoiding visceral manipulation, the Weaver approach offers a valuable alternative to traditional combined or anterior approaches. Future studies involving larger case series and extended follow-up periods are necessary to better define selection criteria, long-term spinal outcomes, and recurrence rates associated with this technique. Additionally, comparative studies between the Weaver and Wiltse approaches could further refine surgical decision-making for paravertebral nerve sheath tumors.

## Data Availability

The original contributions presented in the study are included in the article/supplementary material. Further inquiries can be directed to the corresponding author.
